# Medical Opinion and Movement

**Published:** 1909-01-30

**Authors:** 


### Medical Opinion and Movement.

Reports and expressions of opinion more or less laudatory have already made the medical profession familiar with the Keating-Hart method of fulguration of cancerous growths. Drs. Laquerrière and Delherm discourse on the subject in the *Journal de Mèdecine.* They have had the opportunity of seeing a number of patients, previously declared inoperable, who have been relieved of their cancers for some months and are to all appearances in a perfect condition of health. After removal of the growth by ordinary surgical means, and while still under an anæsthetic, it will be remembered, electric sparks of high frequency are allowed to play upon the site of the growth from an Oudin resonator. The burning effect of the sparks is prevented by a strong current of carbonic acid gas under pressure. The immediate effect is to stop all hæmorrhage and allow of a minute examination for further traces of the growth. Some other effects noted by these observers are entire absence of pain after the operation, a very profuse lymphorrhœa which soaks through all dressings, and which, as they suggest, possibly helps to carry off any remaining germs of growth in the tissues, and a very rapid cicatrisation giving the best æsthetic results and also a quick convalescence. In the case of cancer of the rectum Keating-Hart is able to remove the growth without the usual preliminary colotomy.

---

An interesting report appears in the *Progrès Médical* by Dr. J. Tanton on the treatment of two cases of tetanus by injections of anti-tetanic serum and intraspinal injections of magnesium sulphate solution. One case of considerable severity succumbed, but a definite, though temporary, cessation of the spasms followed the magnesium sulphate injections. The second less acute case recovered completely. Dr. Tanton offers some valuable suggestions in commenting on these cases. In the first place, there has been some discussion as to whether an isotonic or hypertonic solution of magnesium sulphate should be used, and it has been suggested that an isotonic solution is safer and will prevent the severe headache and other accidents likely to follow the injections. Dr. Tanton used at first an isotonic solution, and the injection was followed by severe headache which lasted till the spasms recurred. Afterwards he used a 25 per cent. solution and considers this more efficacious. He regards the treatment as of value, but only symptomatic in relieving the spasms by opposing the effect of the tetanus virus on the central nervous system. With regard to the anti-tetanic serum, he strongly advocates intravenous injections as well as local injection at the seat of the original lesion. Henderson Smith has shown that slowly diffusible substances, such as the anti-bodies in general, enter very slowly into the general circulation when injected subcutaneously. According to this authority such antibodies take 15 to 30 hours to reach a maximum strength in the blood after intraperitoneal injection, and two to three days after subcutaneous injection. To choose, therefore, the subcutaneous method instead of the intravenous means a loss of two or three days before the full action of the serum is reached.

---

Under the heading of "An Apology for Cannibalism" a somewhat amusing, and certainly suggestive, article is contributed by Dr. P. Carnot to *Le Progrès Médical*, in which he seeks to show that the barbarous custom of living on one's fellow-creatures is at least physiologically correct. Quite recently Busquet has studied the nutritive effect of feeding frogs with the flesh of other frogs, as compared with frogs fed on veal or mutton. He finds that the frogs fed thus in a cannibalistic fashion show a greater increase in weight in a given time than the control frogs fed on veal and mutton, although they actually received a smaller proportion of proteid. Moreover, this result accords with many other well-known physiological facts. In regard to sera, for instance, there is a great difference in their assimilation and toxicity according as the serum injected is derived from an animal of the same species or of a different species. Dr. Carnot has himself experimented with skin-grafts. If a piece of black skin from a parti-coloured animal be grafted on to a white skin the development of the black graft can be easily watched. The author finds that if the graft is from the animal itself it develops and proliferates much more rapidly than if it is from another animal, even of the same species, and grafting between animals of the same litter is more successful than between animals of different litters, and they may fail altogether between animals of different species even though nearly related. It appears, therefore, that there is a very definite specificity in the animal tissues, and whether it be tissues grafted, or sera injected, or nutriment absorbed, the adaptation is the more or less perfect according as the tissue, serum, or nutriment is derived from the organism itself or from an organism of the same species or of a different species.

---

Dr. J. Lehrmitte, whose important contributions to *La Semaine Médicale* on nervous diseases have frequently received notice in these columns, discusses the pathology of certain amyotrophies which not infrequently accompany the ordinary symptoms of tabes dorsalis. It appears that these muscular atrophies and pareses are not all referable to one common pathological lesion. There are, in the first place, the atrophies of the muscles, secondary to the arthropathies which occur in the course of the disease, and which are known as Charcot's joints. From these are to be distinguished certain primary atrophies which the author separates into two kinds. The one kind are distinguished clinically by developing late and slowly; they are symmetrical and not accompanied by the reaction of degeneration. They usually commence in the lower extremities, and especially attack the interosseous and antero-external groups of muscles of the leg, leading to deformities and contractures. The muscular atrophies of the second category attack the muscles of the trunk, the scapular girdle, the tongue, and the palate. They may assume the Aran-Duchenne type, they develop more rapidly, and are accompanied by the reaction of degeneration and fibrillary contractions. The importance of this distinction lies in the fact that the pathological lesion underlying this first group of atrophies appears to be localised in the peripheral nerves, and is regarded as an inherent factor in the development of the disease. On the other hand the second group, according to the latest pathological research of Raymond, Wilson, and others, owes its origin to changes in the motor nerve cells of the spinal cord and bulb, and there is reason to believe that these degenerative changes are of a syphilitic origin, and although developing *pari passu* with tabes, are in a sense independent of it. The distinction has, therefore, a therapeutic significance. These atrophies of syphilitic origin, if taken at a sufficiently early stage, may yield to specific treatment, or at least their progress may be arrested.

---

Dr. Bucura has examined the milk of a number of women a week after confinement with a view to determining the drugs which are excreted in the milk when administered in medicinal doses, and the relation which this excretion bears to the method of administration of the drug. Iodides, salicylates, ether, antipyrin, bromides, arsenic, and urotropine are so excreted. Mercurial preparations do not all behave in the same manner, and results are different according as the drugs are administered by the mouth, the skin, or subcutaneously. Calomel given by the mouth in dose of 30 to 40 centigrams can be recovered from the milk. If the same salt be given as a suppository the result is the same. No mercury can, however, be found in the milk, if the drug be given by inunction, or by subcutaneous injection. Quinine, phenacetin, hydrastis, salol, codeine, naphthol, carbonate of lithium, digitalin, and sulphur if used in the form of an ointment cannot be discovered in the milk. Moreover, the following purgatives cannot be isolated in the milk after inunction: rhubarb, senna, cascara, and tartaric acid.

---

The lungs of town-dwellers are found on post-mortem examination to be covered with black specks, particularly in the sub-pleural region, where the dust absorbed by the organ is deposited. These specks on casual examination would seem to be disposed without any particular order, but in reality they correspond to the disposition of the lymphoid tissue, which plays a great part in the resorption and fixation of dust in the lungs and pleura. Julius Arnold has contributed a very complete study of these lymphoid collections to Virchow's Archives. In man and animals he has found them in the pulmonary parenchyma, under the visceral pleura, and even in the mediastinal pleura. They have also been found in the costal and diaphragmatic pleura. These nodules in the lung are situated in the spaces contained between the pulmonary lobules and, according as their situation is peripheral or central, are star-shaped or cuneiform, or flattened beneath the pleura. Those of the parietal pleura are raised up like the nap of a cloth. They consist of an irregular heaping up of round cells, held together by a thin layer of connective tissue, and are developed along the length of a vessel. Sometimes small capillaries run through the nodules, these capillaries being found more frequently in the nodules of the parietal pleura than in those of the lung. In children they can be found, but are invisible to the naked eye owing to the absence of dust, rendering them indistinguishable from surrounding tissues. Sometimes these nodules attain great size and may become pedunculated.

---

Some authors have considered these lymphoid nodules as pathological growths—but this is not the truth. They are normal elements of the respiratory apparatus, and their *rôle* is to serve as filters between the respiratory tissues and the nearest lymphatic ganglia. An examination of preparations from subjects of different ages will show this to be the case. They exist in the child; dust and pigment being deposited at a later date. They can be found in the aged who have always lived in country air, and here again they are free from deposit. Arnold's experiments have, in addition, proved the nature of their functions as fixers and absorbers of dust. He took a number of rabbits and exposed them for a time to an atmosphere laden with dust, and immediately afterwards killed half of them. He found their lungs full of solid particles. The remaining rabbits were placed in pure air and killed some time later. No dust was found in the respiratory tissues of these animals, but much was found in the nodules of lymphoid tissue. This disposition of lymphoid tissue must therefore be considered normal and regarded as the first lymphatic barrier to the entry of foreign bodies into the circulation.

---

Schanz and Stockhausen have recently published in V. Graefe's Archiv f. Ophtalmologie a systematic study of various illuminants from the old oil lamp to the present-day electric arc lamp. They have been able to show that in proportion as the illuminants have increased in power, so have they given off an increased proportion of ultra-violet rays. Illuminants in actual use at present may be arranged in the following order as regards their content in ultra-violet rays, and, in consequence of this, in their order of harmfulness to the eyes: (1) Paraffin, (2) gas, (3) ordinary electric lamp, (4) incandescent gas lamp, (5) acetylene, and (6) electric arc lamp. It is unnecessary to multiply examples of the harmful influence exerted on the eyes by the ultra-violet rays such as electric ophthalmia following short-circuiting of currents at high tension, exposure to Finsen rays for a long period, etc., etc., but the ordinary artificial illuminants in constant use are by no means free from harmfulness by reason of their excessive content of ultra-violet rays. Many people are unable to work at night with artificial light because of eye fatigue, and redness and watering of the eyes if an attempt be made to do so. But it is not only the external portions of the eye which suffer, for Birch-Hirchfield has been able to show experimentally that ultra-violet rays produce in the retina of animals a chromatolysis of the ganglion cells and vacuolisation of their protoplasm.

---

It is true that the crystalline lens protects the retina from these rays, but Hirchfeld and Hess have succeeded in producing opacities in the lens by prolonged action of the rays upon this organ. Hence it would seem possible that these rays may be a factor in the production of senile cataract. In order to guard against the dangers of our latter-day illuminants, Schanz and Stockhausen have studied the spectral modifications produced by the interposition of different kinds and colours of glass. Frosted and opalescent glass has no effect on the transmission of these rays. Green and blue glass, formerly recommended as preservatives for the eyes, easily allow of the passage of ultra-violet rays; smoked glass, while preventing their passage, at the same time cuts off most of the luminous rays. Yellow glass alone cuts off the rays without injuring the luminosity, but this power varies in different kinds of glass. It is, however, not essential that all the ultra-violet rays should be cut off, as when not present in excess their action is beneficial to life.

---

SIR DAVID BRUCE and Captain H. R. Bateman publish in the *Journal of the Royal Army Medical Corps* their experiments made with a view to determining whether trypanosomes have an ultra microscopical stage in which they are capable of passing through a Berkefeld filter. It is known that the undiscovered virus of South African horse sickness can do this, and it has been reported by Plimmer, Moore, Breinl, and MacNeal that Trypanosoma gambiense and T. lewisi in culture can do the same. As a result of a series of experiments on five rabbits and eleven white rats, they find that the serum of similar animals infected with nagana is not pathogenic after passage through a Berkefeld filter. Further, the filtered blood of rats suffering from nagana and surra, which have been treated with antimony until all trypanosomes have disappeared from their blood and tissues, is similarly non-infective. The same holds good of Trypanosoma lewisi cultured on blood agar. It is therefore concluded that there is no stage in the development of trypanosomes in which they are small enough to pass through the pores of an ordinary Berkefeld filter. The filters used were of the ordinary pattern, and they were proved, before use with the infected sera, to be capable of stopping the passage of Micrococcus melitensis.

---

A VERY unusual case of obstructed labour is reported from New York by Dr. L. C. Love. The patient was an Italian woman, a primipara twenty-five years of age, and was three days in labour under the care of a midwife before being seen by a medical man. A hard mass was found obstructing the advance of the fœtal head, which was at first diagnosed as an exostosis on the inner side of the symphysis pubis. However it was possible slightly to move the mass, so the alternative of an impacted vesical calculus was adopted. The patient had never had any symptoms suggestive of calculus, but the diagnosis was confirmed by the subsequent course of events. The bladder was incised per vaginam; but the calculus could not be extracted, because the uterus was so tightly contracted upon the fœtus that the head of the latter could not be pushed up sufficiently far to permit of the release of the stone. So an abdominal Cæsarean section was performed, with complete success. A living child was secured, of seven pounds weight, and the calculus then extracted through the previously made vaginal wound: the stone proved to be an inch thick and nearly three inches long, and weighed four ounces. Both wounds healed by first intention, and mother and child ultimately did well, though the former developed lobar pneumonia during the puerperium, a complication which in view of the protracted labour and the operative proceedings was a very grave one.

---

IN an article on lupus in this month's *British Journal of Tuberculosis*, Mr. Adler, of the skin department of the London Hospital, mentions a curious point regarding the complication of this disease with pulmonary tuberculosis. During the last seven years there have been treated in the Finsen Light clinic of this institution 473 female patients. Three of them died of rapid consumption, and in each case, although naturally local treatment was suspended, the skin lesion healed up of itself. Conversely, it has been noticed that sudden unexplainable improvement of lupus is sometimes followed by rapid worsening of the general condition, with death from acute tuberculosis. The explanation advanced is that cutaneous healing results from flooding of the tissues, including the skin, with anti-bodies of tuberculosis—and it will be remembered that the few successes of Koch's old tuberculin treatment, in which massive doses were used, were largely cases of lupus.

---

A FORM of acute tuberculosis in children was described by Landouzy at the American Tuberculosis Congress. It is characterised by continuous fever, quick pulse, and splenic enlargement, but lacks at first local indications. The points of difference from enteric fever are the great irregularity of the pyrexia and the high pulse-rate, besides, of course, negative Widal reaction and absence of rose spots. The condition clears up gradually, signs of deposits in the lungs, pleura or mesenteric glands then becoming evident. The serum test for tubercle may be obtained, and a similar state of "tuberculous sepsis" can be produced artificially in animals. Landouzy has made several generalisations on tuberculosis, among others that children who have undergone tracheotomy often die later of consumption, and that almost all emphysema is of a tuberculous nature.

